# One-step synthesis of highly fluorescent carbon dots as fluorescence sensors for the parallel detection of cadmium and mercury ions

**DOI:** 10.3389/fchem.2022.1005231

**Published:** 2022-09-30

**Authors:** Qiren Tan, Xiaoying Li, Lumei Wang, Jie Zhao, Qinyan Yang, Peng Sun, Yun Deng, Guoqing Shen

**Affiliations:** ^1^ School of Agriculture and Biology, Shanghai Jiao Tong University, Shanghai, China; ^2^ YunNan (Dali) Research Institute of Shanghai Jiao Tong University, Dali, Yunnan, China; ^3^ Shanghai Pudong Agriculture Technology Extension Centre, Shanghai, China

**Keywords:** carbon dots, cadmium ion, mercury ion, quantitative detection, food safety

## Abstract

Cadmium (Cd^2+^) and mercury ions (Hg^2+^) are essential for the quality control of food samples because of their serious toxicity to human health, but the effective and simple strategy for their parallel detection remains challenging. In this paper, a rapid and simple parallel detection method for Cd^2+^ and Hg^2+^ was developed using carbon dots (CDs) as fluorescent sensors. A one-step hydrothermal method with a single precursor l-arginine as both the carbon and nitrogen sources was employed to prepare nitrogen-doped CDs (N-CDs). N-CDs exhibited a uniform particle size and excitation-independent fluorescence emission. The maximum emission wavelength of N-CDs was observed at 354 nm with the excitation wavelength at 295 nm. The quantum yield of N-CDs reached as high as 71.6% in water. By using sodium diphosphate and phytic acid as masking agents, the fluorescent sensor can be quenched by Cd^2+^ and Hg^2+^ in the linear range of 0–26.8 μM and 0–49.9 μM within 5 min. Other common ions in farm products showed no significant effect on the fluorescence intensity of the sensing system. The results demonstrated that the sensing system had good selectivity and sensitivity for Cd^2+^ and Hg^2+^. The detection limits for Cd^2+^ and Hg^2+^ were 0.20 and 0.188 μM, respectively. In addition, the fluorescent sensor had been successfully applied for the detection of Cd^2+^ and Hg^2+^ in fruits and vegetables, and the recoveries were 86.44–109.40% and 86.62–115.32%, respectively. The proposed fluorescent sensor provides a rapid, simple, and sensitive detection method for Cd^2+^ and Hg^2+^ in food samples and thus a novel quantitative detection method for heavy metal ions in foods.

## Graphical Abstract

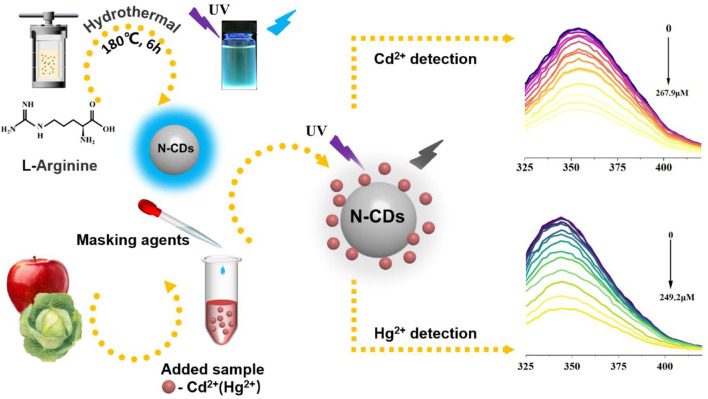

## Highlights

1) Nitrogen-doped carbon dots (N-CDs) were prepared by hydrothermal treatment of l-arginine.
2) N-CDs showed selective and sensitive determination properties of Hg2+ and Cd2+.
3) N-CDs were successfully applied to apple and cabbage samples.

## Introduction

Cadmium (Cd) and mercury (Hg) are the most problematic heavy metals, and both compounds are genotoxic. They cause several health hazards, even at low concentrations, through food (Chukwuemeka-Okorie et al., 2018). Toxic heavy metals can inhibit many enzymatic activities, which can cause serious damage to the reproductive, cardiovascular, and nervous systems (Zhai et al., 2015). Cd is very toxic and probably carcinogenic at low concentrations because of its very long half-life (Xu et al., 2020). Hg is a global health threat and causes impairments in the human nervous system (Karthikeyan et al., 2021). Many terrible diseases such as Minamata and Parkinson’s disease are related to the overexposure and excessive accumulation of mercury (Cai and Wang, 2022). The International Codex Alimentarius Commission stipulates that the maximum residue limit for Cd in vegetables is 0.1 mg kg^−1^, and China sets the limit for Hg at 0.01 mg kg^−1^. Therefore, low concentrations of Cd and Hg ions should be detected for remediation and mitigation and thus prevent serious health problems.

Various analytical methods have been reported for the determination of Cd and Hg ions, including atomic absorption spectroscopy (AAS) (Bagheri et al., 2012), fluorescence spectroscopy (FS) (Prestel et al., 2000), and inductively coupled plasma mass spectrometry (ICP-MS) (Peng et al., 2017). Although these methods have very good sensitivity and accuracy, most of them require high costs, highly trained analysts, and rigorous experimental conditions (Malik et al., 2019), thus restricting their popularity for their field application. Carbon dots (CDs), as novel fluorescent nanocarbon materials with many advantages such as low toxicity, good selectivity, high sensitivity, and stable photostability, have received intense attention in the field of heavy metal detection (Chen et al., 2020). Generally, CDs could interact with heavy metals differently, resulting in quenching mechanisms, such as static, dynamic, inner filter effect, and fluorescence resonance energy transfer. The photoluminescence changes allow the quantification of heavy metal concentrations (Ng et al., 2021). Nevertheless, in many cases, CDs have multi-step and time-consuming synthesis procedures (Wei et al., 2012), need a final functionalization/passivation on their surface (Tian et al., 2009), or require the utilization of sophisticated instruments for preparation (Hu et al., 2009). In the field of food safety supervision, a rapid, simultaneous, and qualitative detection of multiple pollutants is easy for scale-up, exhibiting very promising practical application. Therefore, methods with easy preparation, high selectivity, and quick response are aimed.

To date, many synthetic methods have already been developed to perfect CDs’ functionality for various applications. Among these methods, the hydrothermal synthesis route based on the water system is among the simplest and most cost-effective methods owing to its cheap apparatus, simple manipulation, low energy consumption, good selectivity, and preparation can be achieved in a single step without complex control (Liang et al., 2013; Sun et al., 2022). Furthermore, heteroatom doping can effectively improve the fluorescence performance of CDs (Jana et al., 2019). Notably, nitrogen has a similar size and structure to carbon, and its lone pair electrons easily bond to carbon-based materials, thus remarkably improving the optical properties of CDs (Sun et al., 2021; Xu et al., 2021; Wang et al., 2022).

Apart from the synthesis method, an appropriate carbon precursor should be considered. Many raw materials, including less harmful organic chemicals, solvents, or natural precursors, are used to produce CDs (Wang et al., 2021; Xu et al., 2022). Considering the natural or less harmful chemicals as a precursor for CDs synthesis, the use of amino acids is an option. Amino acids are rich in carboxyl and amino functional groups and are one of the ideal carbon sources for CDs preparation. l-arginine is often used as a carbon source by scientific researchers for the preparation of unique CDs because of its highest nitrogen content within 20 essential amino acids. In most of these studies, CDs-based sensors are prepared for the detection of single heavy metal ions rather than multiple targets (Yarur et al., 2019). Li et al. (2015) prepared nitrogen and sulfur co-doped CDs through a facile one-step microwave-assisted method and used the CDs as fluorescence probe for Hg^2+^ detection. Besides, these studies are focus on the detection of Cd^2+^ and Hg^2+^ in aqueous solution based on carbon dots, while few reports were related to the detection in food samples by using carbon dots. Cd^2+^and Hg^2+^ frequently coexist in many environmental samples with potential danger to humans through the food chain. Therefore, new methods for synthesizing CDs should be developed for the simultaneous, rapid, and qualitative detection of Cd^2+^and Hg^2+^.

In the present work, we report novel N-doped CDs (N-CDs) prepared by an easy one-step hydrothermal method with l-arginine as precursors. The fluorescent property of N-CDs was found independent of the excitation wavelength and sensitive to Cd^2+^ and Hg^2+^. N-CDs showed remarkable sensitivity and selectivity when used as the fluorescent sensor for Cd^2+^ and Hg^2+^ with the masking agents. The application of the sensor for the detection of heavy metals in food was improved by applying it for the detection of food samples with a good recovery ratio. Notably, l-arginine is commercially available and could be used to synthesize CDs directly. The complex synthesis of starting materials was not needed, and the synthesis procedure was easy to carry out. To the best of our knowledge, a sensor that can simultaneously detect Cd^2+^ and Hg^2+^ is rare, and research in the detection of Cd^2+^ and Hg^2+^ in food samples by using carbon dots is also sparse.

## Materials and methods

### Materials

L-arginine and standard reserve solutions of Cd^2+^ (892.9 μM), and Hg^2+^(498.5 μM) were obtained from *Aladdin* (Aladdin, Shanghai, China). Potassium chloride (KCl), sodium chloride (NaCl), magnesium chloride (MgCl_2_), calcium chloride (CaCl_2_), manganese chloride (MnCl_2_), zinc chloride (ZnCl_2_), aluminum chloride (AlCl_3_), nickel chloride (NiCl_2_.6H_2_O), sodium nitrate (NaNO_3_), sodium nitrite (NaNO_2_), sodium dihydrogen phosphate (NaH_2_PO_4_), sodium sulfate (Na_2_SO_4_), 1,10-phenanthroline, Rochelle salt, EDTA, sodium diphosphate, phytic acid, potassium iodide, acetate buffer, and trisodium citrate dihydrate were obtained from Sinopharm Chemical Reagent (Shanghai, China). All the reagents and solvents of analytical grades were used without further purification. All the experiments were carried out using deionized water.

### Preparation of N-CDs

N-CDs were synthesized using the typical one-step hydrothermal method. In short, 0.2 g of l-arginine was dissolved in 40 ml of deionized water and sonicated for 20 min. The mixture was transferred to the reaction kettle, heated at a given temperature for a given time, and cooled at room temperature. Afterward, the solution was filtered through a 0.22 μm filter membrane to remove the precipitate. Finally, the obtained solution was then diluted for 20 times with deionized water. After freeze-drying, the purified N-CDs were obtained. The N-CDs showed bright blue luminescence under a UV illuminator.

### Characterization of N-CDs

Fluorescence was detected using Infinite M200 Pro (Tecan, Switzerland). UV-vis spectroscopy was carried out using Cary 60 UV-Vis (Agilent, America). FT-IR spectroscopy was carried out using Nicolet 6,700 (Nicolet, America). X-ray photoelectron spectroscopy (XPS) was conducted using AXIS UltraDLD (Shimadzu, Japan). TEM was performed using Talos F200X G2 (FEI, America). HRTEM images were collected using a JEM-2100F (JEOL, Japan). XRD was performed using a D8 ADVANCE Da Vinci (Bruker, German). The Raman spectra were determined using the inVia Qontor Confocal Raman microscope (Renishaw, UK). The pH-dependent photoluminescence sensitivity of N-CDs was studied at different pH values by using different buffer systems (citrate buffer, pH 5.0–11.0). N-CDs were irradiated with a 365 nm UV lamp to investigate their photostability against photobleaching. Quinine sulphate in 0.1 M H_2_SO_4_ was used as the standard sample to measure the QY of N-CDs.

### Selectivity of N-CDs and screening of making agents

Approximately 10 mg L^−1^ of K^+^, Ca^2+^, Na^+^, Mg^2+^, Mn^2+^, Zn^2+^, Al^3+^, Hg^2+^, Ni^2+^, and Cd^2+^ ions were added into 100 μL of N-CDs solution to study the selectivity of N-CDs. To screen the appropriate masking agents, we added 20 mg of various masking agents to 100 ml of 446.45 μM Cd^2+^ (Hg^2+^ ions, 249.25 μM), and then vortexed for 30 s. Approximately 50 μL of the supernatant, 20 μL of N-CDs, and 230 μL of citrate buffer (pH 7.0) were mixed, and the fluorescence intensity was measured.

### Detection of Cd^2+^ and Hg^2+^

The detections of N-CDs toward Cd^2+^ and Hg^2+^ were evaluated in the aqueous solution (pH 7.0) by using Cd^2+^ and Hg^2+^ concentrations at room temperature. For Cd^2+^ and Hg^2+^ sensing, 180 μL of citrate buffer (pH 7.0), 20 μL of N-CDs solution, and 100 μL of different concentrations of Cd^2+^ and Hg^2+^ ions were mixed and incubated at room temperature for a certain time. Then, the fluorescent intensity was tested.

### Detection of Cd^2+^ and Hg^2+^ in food samples

Apples and cabbages were obtained from a local supermarket to verify the reliability of the detection method. Heavy metal digestions were performed as previously described (Liu et al., 2016). Each of the samples (1 g) was digested in 10 ml of nitric and perchloric acids (9:1) solution and heated on a hot plate to 150°C until brown fumes ceased to evolve, and the mixture was concentrated almost to 1–2 ml. The solution was made to a total volume of 25 ml by using 10% nitric acid after being cooled to room temperature. For real sample detections, a certain volume of standard reserve solutions of Cd^2+^ and Hg^2+^ ions was added to the digestion solution, then the samples were detected.

## Results and discussion

### Preparation for the optimization of N-CDs

**FIGURE 1:**
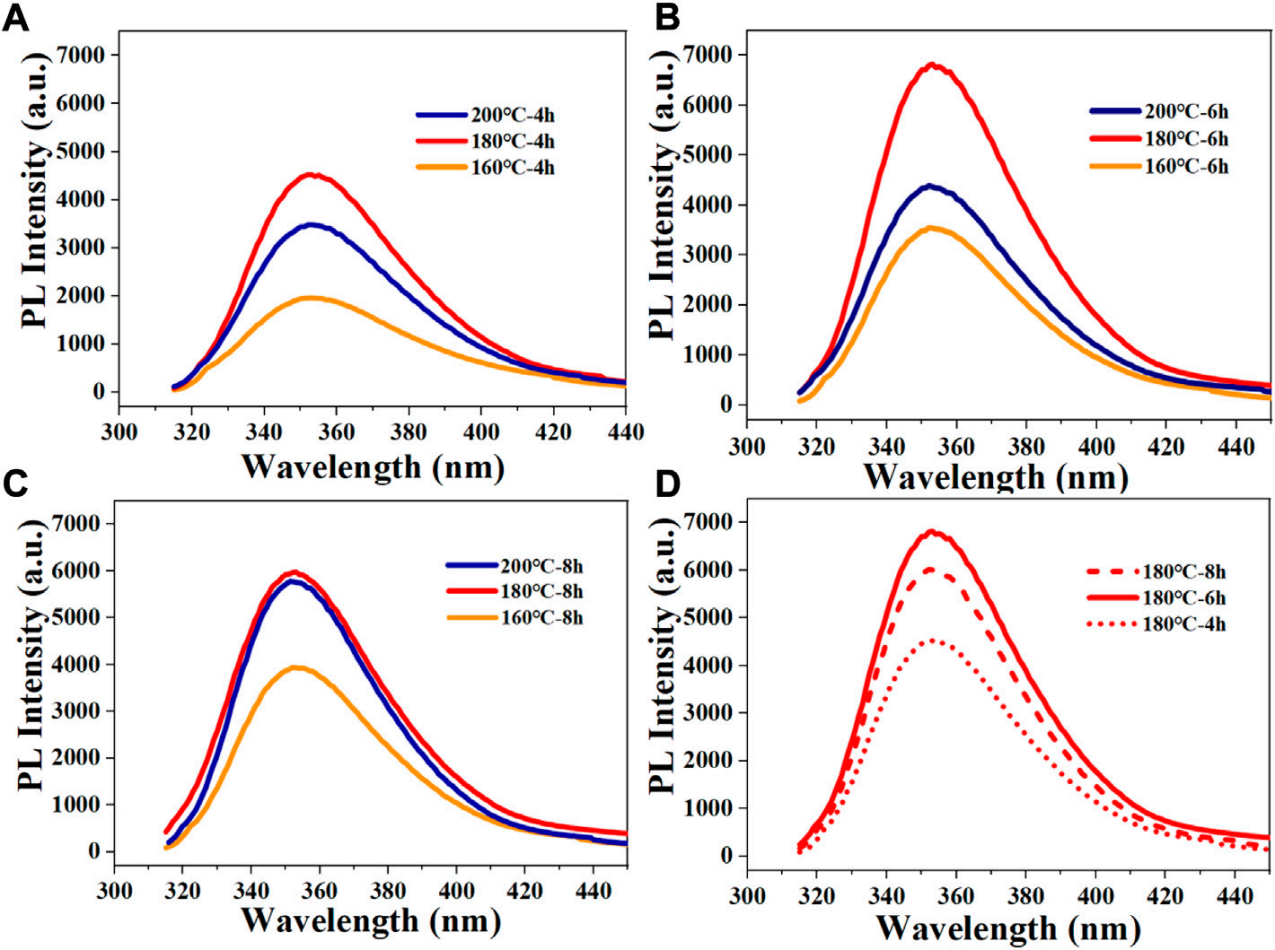
Fluorescence spectra of resultant CDs under different conditions: **(A)** different reaction temperatures with reaction time of 4 h; **(B)** different reaction temperatures with reaction time of 6 h; **(C)** different reaction temperatures with reaction time of 8 h; **(D)** different reaction times with reaction temperature of 180°C.

The preparation conditions of the N-CDs were optimized by varying the prepared temperature and time. As shown in Figure 1, no variation was observed in the shape of the fluorescence emission spectra (excitation wavelength at 295 nm) with different reaction conditions. The optimum temperature was determined by keeping the reaction time constant (4, 6, and 8 h) and changing the temperature. The results showed that the fluorescence intensity of N-CDs increased first and then decreased with increasing temperature, indicating that the reaction temperature played an important role in influencing the optical properties of N-CDs (Figures 1A–C). The strongest fluorescence intensity under prepared temperature is 180°C, which was selected as the best prepared temperature. Then, the most efficient reaction time was determined at 6 h by varying the reaction time while keeping the temperature constant at 180°C (Figure 1D). Therefore, the reaction temperature of 180°C and reaction time of 6 h were determined as the optimal conditions to study their further application in fluorescence sensing.

### Characterization of N-CDs

**FIGURE 2:**
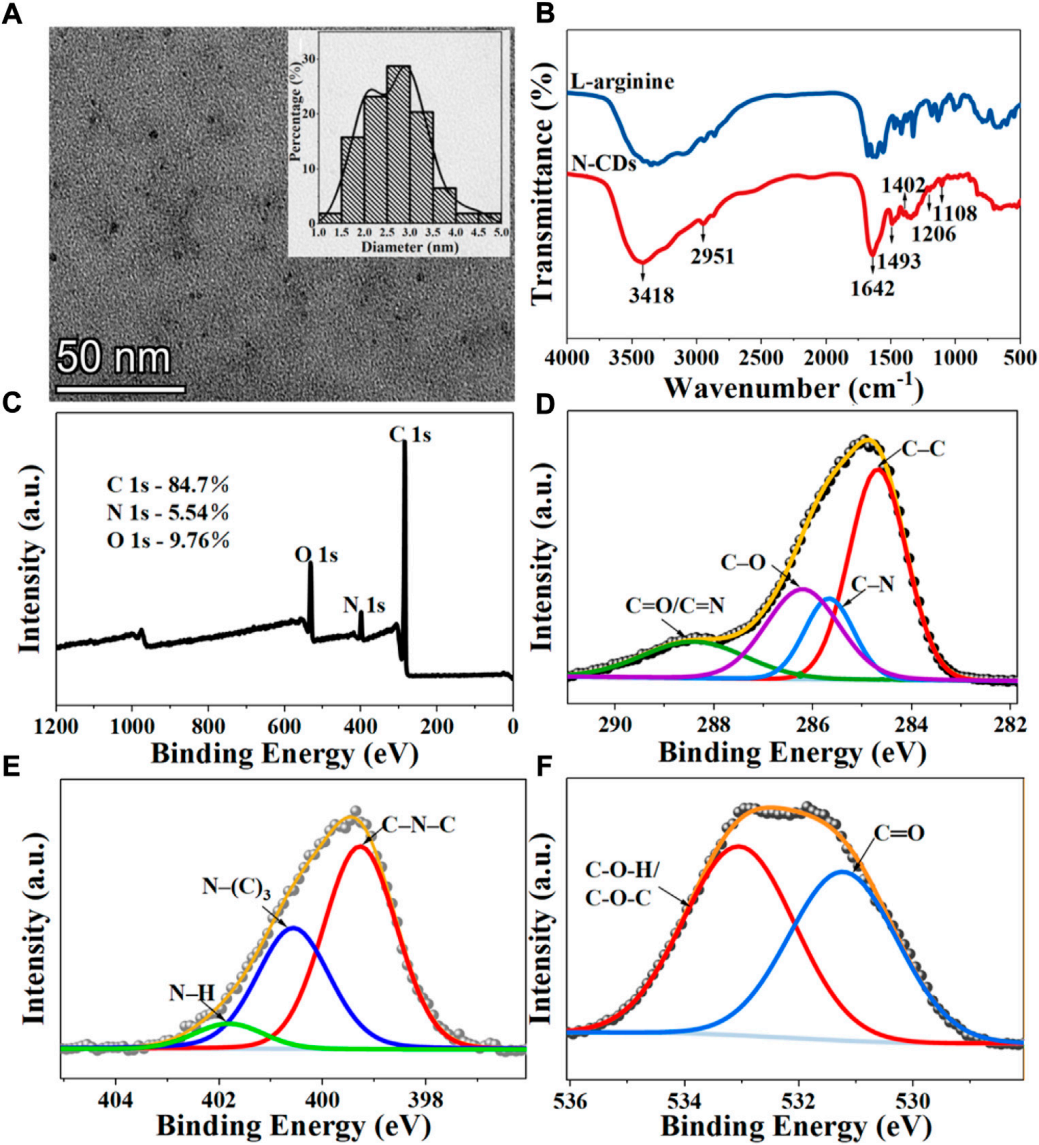
Characterization of N-CDs: **(A)** TEM images. Inset: Size distribution diagrams of N-CDs; **(B)** FT-IR spectra of l-arginine and N-CDs; **(C)** Full survey XPS spectra of N-CDs; **(D)** C 1s XPS spectra of N-CDs; **(E)** N 1s XPS spectra of N-CDs; **(F)** O 1s XPS spectra of N-CDs.

The morphology of N-CDs was observed and characterized by TEM and HRTEM. As shown in Figure 2A, the N-CDs were nearly spherical in shape and had good dispersion. Approximately 110 dots were randomly selected for particle size statistics. The inset in Figure 2A showed that the particle size of N-CDs was in the range of 1–5 nm, with an average diameter of 2.68 ± 0.67 nm, which conformed to the general characteristics of carbon quantum dots. The results of HRTEM were shown in Supplementary Figure S1, it was observed that N-CDs had a discernible lattice structure. The lattice fringes on N-CDs with an interplanar spacing of 0.234 nm could be indexed to the facet of graphitic carbon (100). Supplementary Figure S2 showed the X-ray diffraction (XRD) pattern of the N-CDs. It showed a single broad peak centred at 2θ = 19.68°, which was consistent with the (002) lattice spacing of carbon-based materials with abundant sp^3^ disorder. The Raman spectrum of the N-CDs exhibited two peaks at 1,353 cm^−1^ and 1,586 cm^−1^, corresponding to the D and G bands, respectively (Supplementary Figure S3). The D band is mainly due to the defective vibration of disordered graphite, and the G band is related to the vibration of sp^2^-bonded CDs. The ratio of I_D_/I_G_ is the characteristic of the disorder extent and the ratio of sp^3^/sp^2^ carbon. I_D_/I_G_ in this study was 1.30, which indicated the large number of structural defects in the N-CDs. The above results showed that the nanoparticles formed were mainly amorphous carbon dots and not graphene quantum dots. (Raveendran et al., 2019).

The surface functional groups of the precursor (l-arginine) and product (N-CDs) were compared by FT-IR spectra. Figure 2B presents many similar peaks in the FTIR spectra of l-arginine and N-CDs, which confirmed that most of the nitrogenated and oxygenated functional groups of N-CDs were derived from l-arginine. The most prominent chemical bond vibration peaks of N-CDs were mainly distributed at 3,418, 2,951, 1,642, 1,493, 1,402, 1,206, and 1,108 cm^−1^. Absorption bands at 3,418 cm^−1^ indicated the vibrations of O–H and N–H, and the C–H stretching vibration could be proved by the absorption band at 2,951 cm^−1^ (Zhang et al., 2019). The peak at 1,642 cm^−1^ may indicate the stretching of the C=O groups of a carbonyl group derived from amide and carboxylic acid or C=N stretching vibrations (Wang et al., 2017). The presence of C–N stretching vibration may lead to the appearance of a peak at 1,402 cm^−1^ (Wang et al., 2016). The band that appeared at 1,206 and 1,108 cm^−1^ indicate the stretching vibration of C–O–C (Xu et al., 2014).

XPS analysis was carried out to further study the surface element analysis and functional groups of N-CDs. The XPS full-scan spectra exhibited the presence of peaks for carbon (284.96 eV), nitrogen (399.36 eV), and oxygen (531.86 eV), which was also in good agreement with the results of FT-IR (Figure 2C). The atomic percentages of C, N, and O were 84.7, 5.54, and 9.76%. In the expanded XPS spectra, the C1s peaks at 284.48, 285.25, 286.27, and 288.44 eV shown in Figure 2D can be attributed to carbon in the form of C–C, C–N, C–O, and C=O/C=N (Yang et al., 2014). The N1s spectrum (Figure 2E) shows three peaks at 399.27, 400.57, and 401.82 eV, which are assigned to the C–N–C, N–(C)_3_, and N–H bands (Liu et al., 2017). The XPS spectrum of O 1s exhibited two apparent peaks centered at 531.23 and 533.05 eV, which were related to the C–O and C–O–H/C–O–C groups (Figure 2F) (Prasannan and Imae., 2013).

Based on the above results, the nitrogen-doped CDs from l-arginine were successfully synthesized. N-CDs retained a part of properties and the functional groups with O and N from l-arginine, which endowed their excellent water solubility.

### Optical properties of N-CDs

**FIGURE 3:**
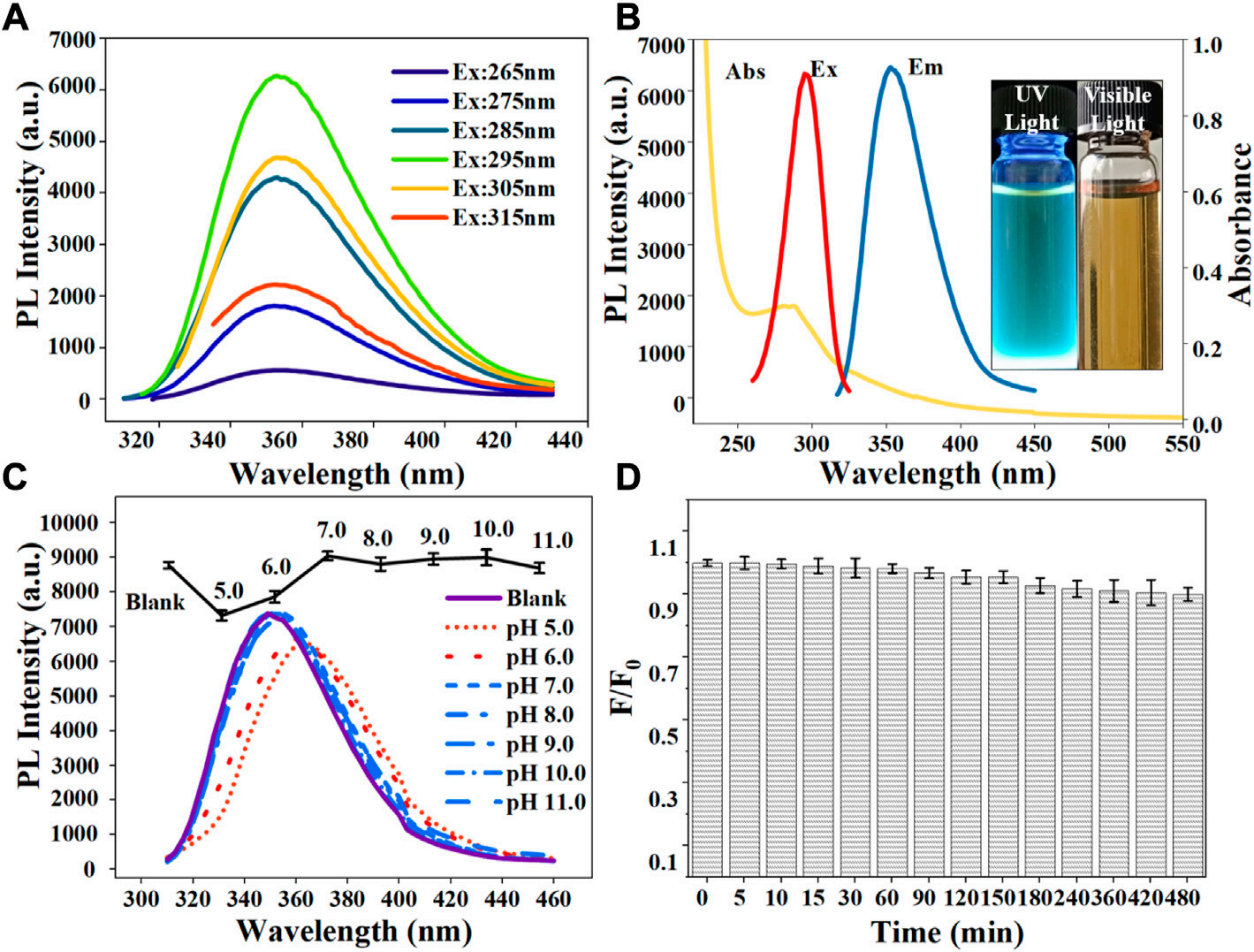
**(A)** Fluorescence emission spectra of N-CDs under different excitation wavelengths ranging from 265 to 315 nm; **(B)** UV–vis absorption, maximum fluorescence excitation, and maximum emission spectra of N-CDs. Inset: photographs of N-CDs solution under 365 nm UV light (left) and visible light (right); **(C)** Effect of pH on the fluorescence intensity of the N-CDs; **(D)** Effect of irradiation time with a 365 nm UV-lamp on the photostability of N-CDs.

The optical properties of N-CDs were characterized in terms of the fluorescence spectra and UV-vis. Figure 3A showed that the fluorescence emission spectra of N-CDs were changed with a series of excitation wavelengths ranging from 265 to 315 nm. The emission peaks of N-CDs at various excitation wavelengths did not shift, and the maximum emission wavelength at different excitation wavelengths remained at 354 nm. The property of excitation-independent emissions was different from most of the previously reported CDs (Atchudan et al., 2020; Tang et al., 2021), which can avoid autofluorescence during their applications. This finding was obtained, because N-CDs had a relatively uniform particle size distribution and localized surface state band structure (Chandra et al., 2017). As shown in Figure 3B, the maximum emission wavelength of N-CDs was located at 354 nm at an excitation wavelength at 295 nm. N-CDs aqueous solution showed a UV absorption peak at 288 nm (Figure 3B), which corresponded to the π–π transition of C=C and the n-π transition of C=O (De et al., 2013). The photographs inserted in Figure 3B show that the N-CDs solution appears bright blue under an ultraviolet lamp with UV (365 nm) and solution is brown in visible light.

Photostability tests were carried out to evaluate the feasibility of N-CDs in real environments. As shown in Figure 3C, the fluorescence intensity of N-CDs was partially affected by pH values. Under neutral non-acidic pH conditions (7.0–11.0), the N-CDs had relatively stable optical properties and exhibited a remarkable fluorescence intensity. Their PL intensity decreased under acidic pH conditions (5.0–6.0) possibly because of the presence of oxygen-containing functional groups such as carboxyl groups on their surfaces. In a strongly acidic environment, carboxyl groups may lead to the formation of hydrogen bonds and obstruct the electron-hole pair recombination, thus destroying the electronic transition of the luminescent center and causing quenching fluorescence intensity (Kundu et al., 2012). While in neutral or basic solutions, the functional groups of N-CDs remained stable. Therefore, in subsequent detection experiments, phosphoric acid buffer solution was used as the solvent to eliminate the influence of pH changes.

The QY was determined at an excitation wavelength of 320 nm using equation (Ma et al., 2017):

$$\Phi_{x}=\Phi_{s}\;\left(A_{s}\;/\;Ax\right)\left(I_{x\;}/\;I_{S}\right)\left(\eta_{X}^{2}/\;\eta_{S}^{2}\right)$$

where s and x refer to the standard sample (quinine sulfate) and the sample to be tested, respectively; *Φ* is QY, *Φ*_*s*_ = 54%; *A* is the absorbance at the excitation wavelength (295 nm); *I* is the integrated area of fluorescence in the emission region at 295–600 nm; *η* is the refractive index of solvent, ***η***_***x***_ **/*η***_***s***_ = 1. All the samples were diluted to ensure an absorbance value of less than 0.10 measured. The QY of N-CDs was calculated to be 71.6%.

Additionally, the fluorescence intensity of N-CDs did not vary obviously after continuous irradiation under a UV lamp for several hours. At 480 min after irradiation, the fluorescence quenching ratio *F*/*F*_*0*_ (*F* and *F*_*0*_ are the fluorescence intensities of N-CDs in the presence and absence of heavy metal ions) of N-CDs did not exceed 10% (Figure 3D), indicating the excellent resistance to photobleaching of N-CDs. Besides, N-CDs showed excellent salt-resistant stability (up to 1.8 mol L^−1^) and long-term storage stability (up to 8 weeks) (Supplementary Figures S4, S5). The effects of different batches, long-term storage and salinity on the stability of N-CDs were also studied. In order to study the reproducibility of N-CDs, different batches of l-arginine were used to synthesize N-CDs. As shown in Supplementary Figure S6, the PL intensities of different batches of N-CDs had no significant change. Hence, N-CDs had a good stability and application prospect for the analysis of complex matrixes as fluorescent probes.

### Selective detection of Cd^2+^ and Hg^2+^

**FIGURE 4:**
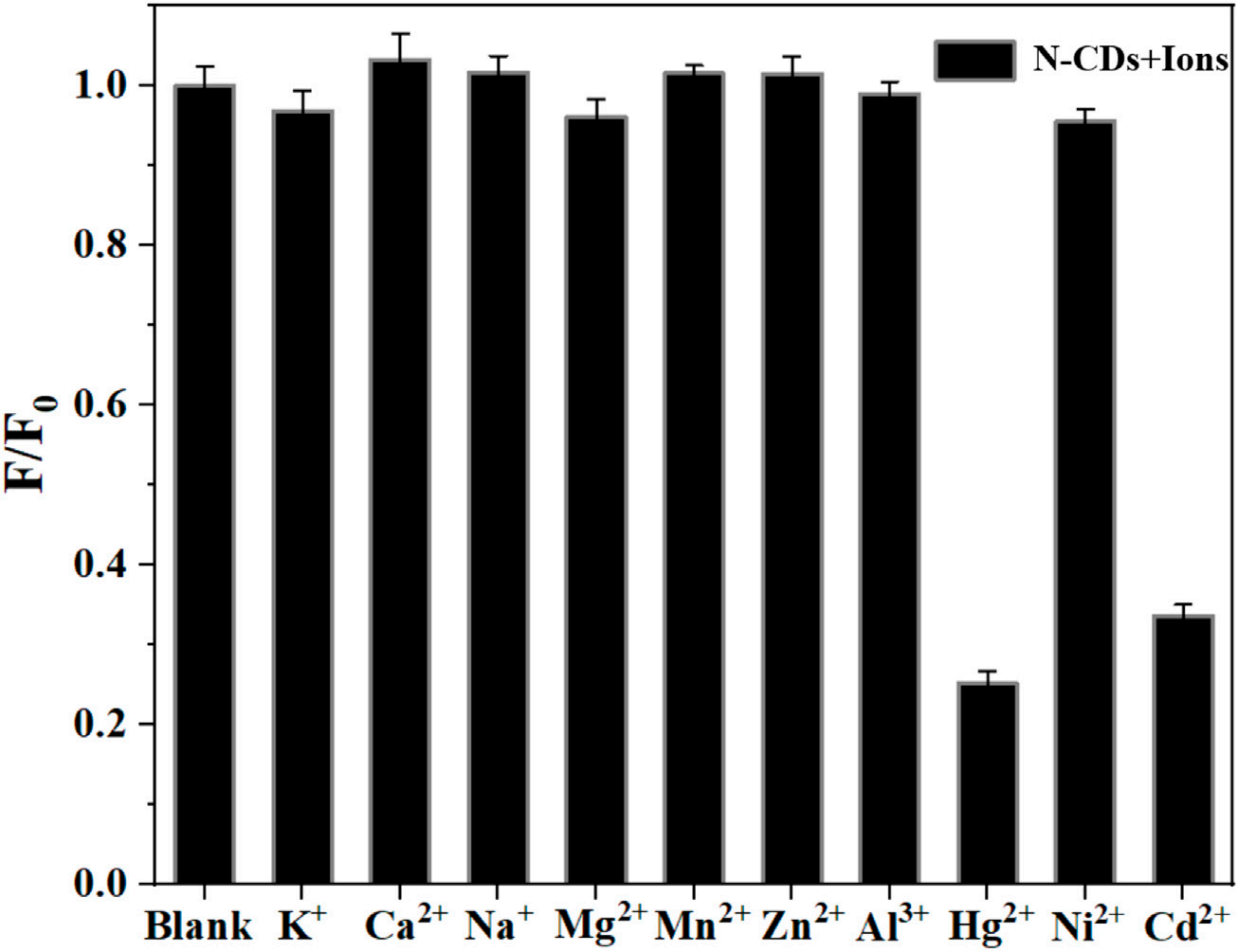
Selectivity of N-CDs toward different metal ions.

Figure 4 shows the effect of various metal ions on the fluorescence performance of N-CDs based on the metal ion-induced quenching of fluorescence. The result indicated that the fluorescence of N-CDs was strongly quenched by Cd^2+^ and Hg^2+^ but not the other physiological or environmentally relevant metal ions, suggesting the potential of N-CDs for the fluorescence detection of Cd^2+^ and Hg^2+^.

**FIGURE 5:**
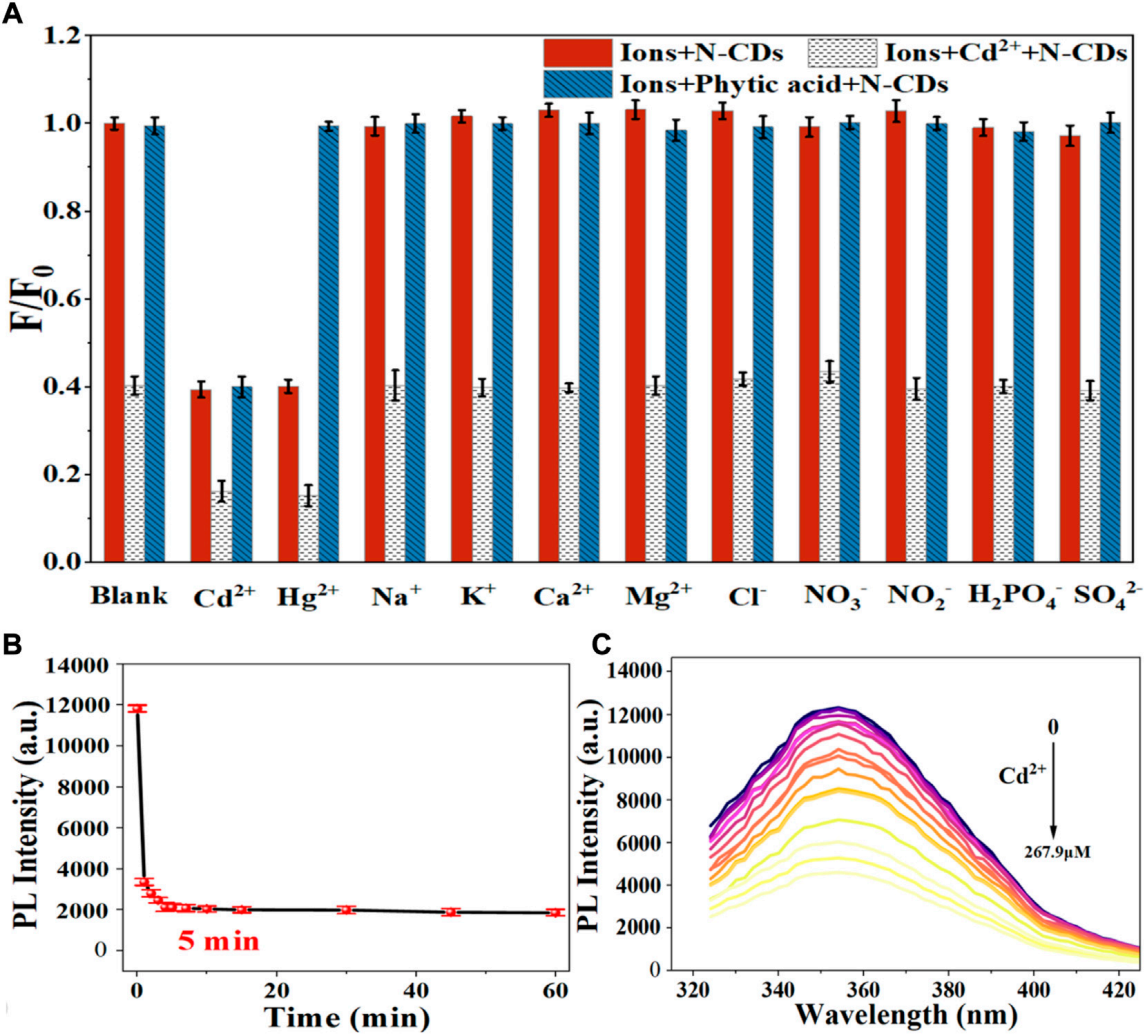
**(A)** Selectivity of the N-CDs detection to Cd^2+^ **(B)** Incubation time of N-CDs with Cd^2+^; **(C)** Fluorescence emission spectra of N-CDs under different concentrations of Cd^2+^.

**FIGURE 6:**
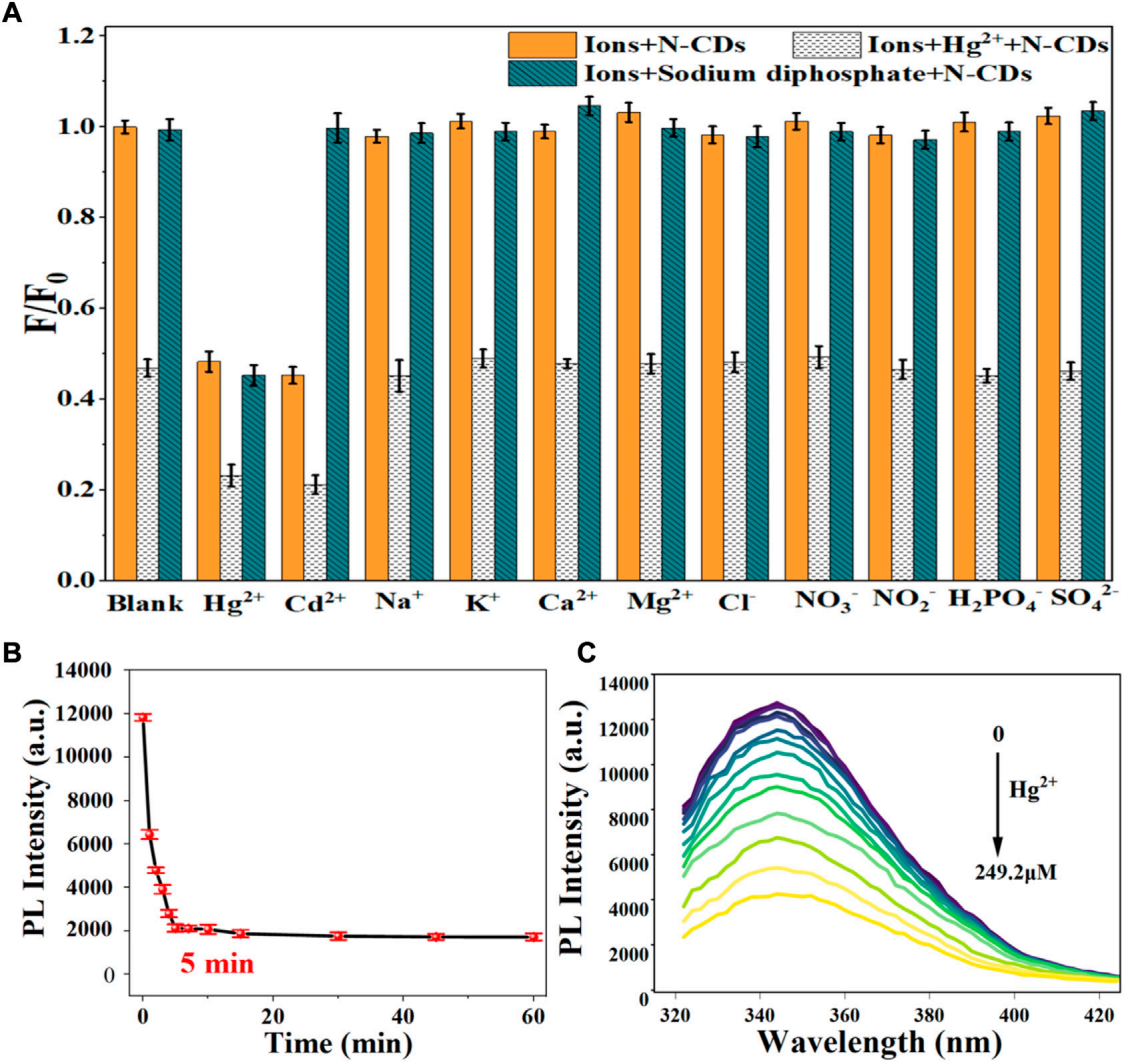
**(A)** Selectivity of N-CDs detection to Hg^2+^; **(B)** Incubation time of N-CDs with Hg^2+^; **(C)** Fluorescence emission spectra of N-CDs under different concentrations of Hg^2+^.

Considering the mutual interference in the detection of Cd^2+^ and Hg^2+^, a series of masking agents were studied to selectively detect only one ion species in Cd^2+^ and Hg^2+^ mixture. As shown in Table 1, various masking agents were screened. 1,10-Phenanthroline and EDTA exerted a strong masking effect on Cd^2+^ and Hg^2+^, while Rochelle salt, potassium iodide, sodium sulphate, acetate buffer, and trisodium citrate dihydrate showed little masking effect. Furthermore, 20 mg of sodium diphosphate and phytic acid can fully mask 44.65 mmol of Cd^2+^ and 26.79 mmol of Hg^2+^, respectively. The addition of the masking agents had no significant effect on the detection of other ions (Figure 5A, Figure 6A). Hence, phytic acid and sodium diphosphate were determined as the masking agents for Cd^2+^ and Hg^2+^ detection, respectively.

**TABLE 1:** Masking agent screening results.

Masking agents	Hg^2+^	Cd^2+^
1,10-Phenanthroline	**−**	**−**
Rochelle salt	**+**	**+**
EDTA	**−**	**−**
Sodium diphosphate	**+**	**−**
Phytic acid	**−**	**+**
Potassium iodide	**+**	**+**
Sodium sulphate	**+**	**+**
Acetate buffer	**+**	**+**
Trisodium citrate dihydrate	**+**	**+**

“+” denotes not masked, “−” denotes fully masked.

### Quenching mechanism investigation

**FIGURE 7:**
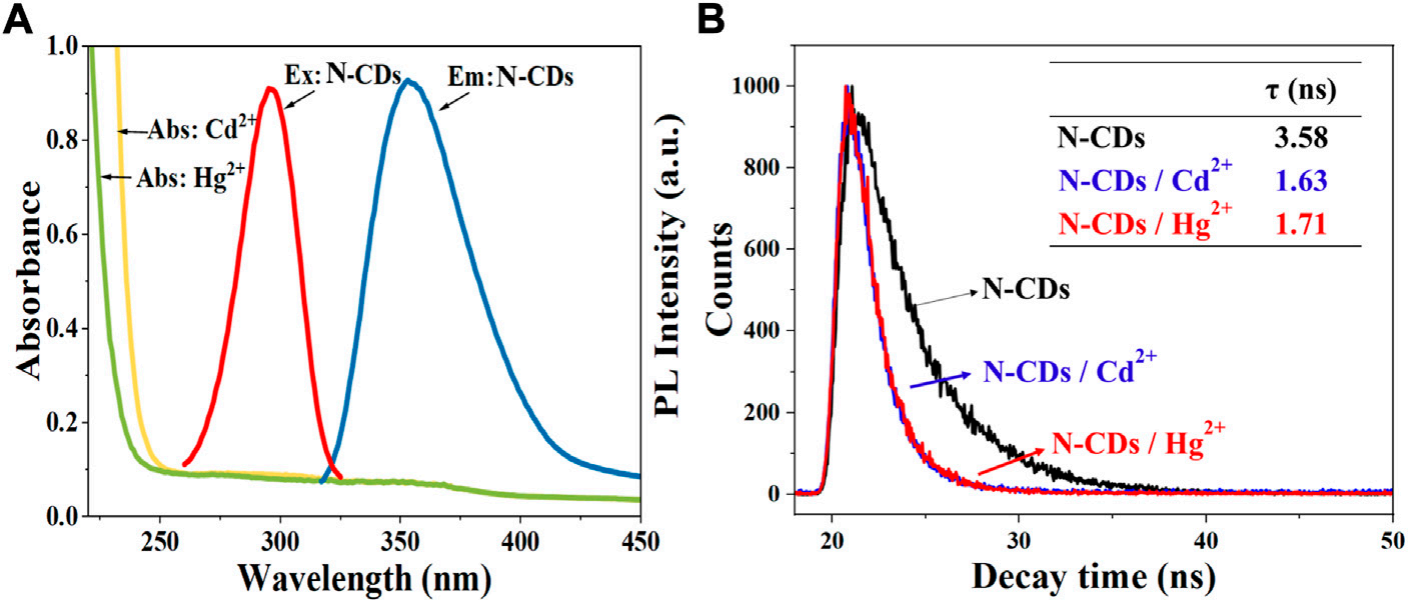
**(A)** UV–vis absorption spectra of Cd^2+^ and Hg^2+^ solutions. Maximum fluorescence excitation and maximum emission spectra of N-CDs; **(B)** The fluorescence lifetime curves of N-CDs, N-CDs/Cd^2+^, and N-CDs/Hg^2+^system.

The fluorescence quenching mechanism of fluorescent materials is usually divided into the inner filter effect (IFE), the static quenching and dynamic quenching, or both of static quenching and dynamic quenching (Iqbal et al., 2016). Generally, IFE occurs when the absorption spectra of the quencher overlap with the excitation and/or emission spectra of CDs (Zheng et al., 2013). As shown in Figure 7A, the absorption of Hg^2+^ and Cd^2+^ did not overlap with the excitation and emission spectrum of N-CDs. Hence, we can infer that the quenching mechanisms of N-CDs/Cd^2+^ and N-CDs/Hg^2+^ were not the IFE.

In order to explore the quenching mechanism, the fluorescence lifetimes of N-CDs, N-CDs/Cd^2+^, and N-CDs/Hg^2+^system were measured. Usually, the fluorescence lifetime of the fluorophore changes in dynamic quenching process, but remains stable in static quenching process (Orte et al., 2013). As shown in Figure 7B, the fluorescence decay curves of the N-CDs presented different route after the addition of Cd^2+^ and Hg^2+^. The single exponential formula could fit the fluorescence attenuation curve very well. And the fluorescence lifetime of N-CDs, N-CDs/Cd^2+^, and N-CDs/Hg^2+^system is 3.58 ns, 1.63 ns, and 1.71 ns, respectively. Fluorescence lifetime changes can be indicative of the dynamic quenching.

As shown in Supplementary Figure S7, the absorption peak of N-CDs at 288 nm showed a red shift effect and the peak intensity was significantly reduced after the addition of Cd^2+^ and Hg^2+^, which confirmed the formation of new complex. This phenomenon could result from the chelating effect between the functional groups of N-CDs and Cd^2+^ (Hg^2+^). The oxygen atom in the–COOH, –OH, and nitrogen atom in the–NH_2_ of N-CDs coordinated Cd^2+^ and Hg^2+^ ions, donating lone pairs electrons into their empty orbitals, and then led to the fluorescence quenching of N-CDs (Lin et al., 2019). Additionally, zeta potential was measured to further elaborate the detection mechanism. The results showed N-CDs had a large potential of -24.67 mV because of the presence of carboxyl and O-functional groups on the N-CDs. After the addition of Cd^2+^ and Hg^2+^, the zeta potential of N-CDs increased to the positive potential of 6.10 and 1.57 mV, respectively, which confirmed that the N-CDs were combined with the Cd^2+^ and Hg^2+^ ions by the ligand-to-metal charge transfer (Wu and Tong., 2019). The results of UV-vis absorption spectra and Zeta were the characteristics of static quenching (Wang et al., 2020).

The Stern-Volmer (SV) equation can be used to describe the quenching mechanism as follows.

$$F_{0}/F=1+K_{q}\;\tau_{0}\left[Q\right]=1+K_{SV}\left[Q\right]$$

Where F_0_ and F are the steady-state FL intensities without and with the presence of a quencher, respectively; K_SV_ is the Stern–Volmer constant, [Q] is the concentration of Cd^2+^ (Hg^2+^) and τ_0_ is the lifetime. According to Stern-Volmer (SV) equation, the SV plot during a single static quenching or dynamic quenching should be a straight line. While it can be seen from Supplementary Figures S8A, S9A that the SV diagram (298K) was not a straight line, suggesting a joint of dynamic and static quenching for the detection mechanism of Cd^2+^ (Hg^2+^) by N-CDs (Li and Tong., 2020).

### Fluorescence detection of Cd^2+^ and Hg^2+^

The sensitivity of N-CDs detection was adjusted to a neutral pH value (7.0) for the optimum detection of Cd^2+^ and Hg^2+^ ions. N-CDs’ anti-interference capability was studied to further check the selective quenching behavior toward other kinds of common disruptors in fruits and vegetables, including Na^+^, K^+^, Ca^2+^, Mg^2+^, Cl^−^, NO_3_^−^, NO_2_^−^, H_2_PO_4_^−^, and SO_4_^2-^ (Webster, 1981; Yang et al., 1998; Henríquez et al., 2010). As shown in Figure 5A, Figure 6A, these ions had little effect on the detection of Cd^2+^ and Hg^2+^ ions. The multiple active sites of N-CDs including carboxylate, hydroxyl groups and abundant amino-groups, may explain the high selectivity of N-CDs to Cd^2+^ and Hg^2+^. Those functional groups led to the interaction between N-CDs and Cd^2+^ or Hg^2+^ through an effective electron transfer process, which caused the fluorescence quenching of N-CDs (Xie et al., 2021). Therefore, most common ions in fruits and vegetables do not interfere with the detection of Cd^2+^ and Hg^2+^ ions. N-CDs were highly selective toward Cd^2+^ and Hg^2+^ ions and had excellent anti-interference even when other metal ions were present.

The quantitative detection of Cd^2+^ and Hg^2+^ were tested under the optimized detection conditions. The incubation time and sensitivity of the detection system were tested. As exhibited in Figure 5B, Figure 6B, the reactions finished rapidly within 5 min. As shown in Figure 5C, Figure 6C, with the graduated addition of the heavy metal ions, the fluorescence spectra of N-CDs decreased gradually. The relationships with Cd^2+^ and Hg^2+^concentration were y = 0.00987x + 1.00851, *R* ^2^ = 0.9909, in the range of 0–26.8 μM, and y = 0.01057x + 0.9965, *R* ^2^ = 0.9981, in the range of 0–49.9 μM, respectively (Supplementary Figures S8B, S9B). The limits of detection (LOD) were calculated following a curve-fitting model (LOD = 3*δ/k*, *δ* represented the intensity standard deviation of the blank samples that was measured 10 times and *k* was the slope of the curve). The LOD values of Cd^2+^ and Hg^2+^ were 0.201 and 0.188 μM, respectively. Compared with other All the above excellent features clearly reveal that the N-CDs as fluorescent probes have great potential for the analysis of Cd^2+^ and Hg^2+^. As shown in Table 2, the QY of N-CDs is higher than most of those in other reports. Compared with other methods for the detection of Cd^2+^ and Hg^2+^, N-CDs showed a wider detection linear range and a higher sensitivity than that reported in other methods.

**TABLE 2:** Comparison of different methods for the detection of Cd^2+^ and Hg^2+^ using CDs as sensing probes.

Precursors of CDs	Ions	QY (%)	Linear range	LOD	Real sample	Reference
Chopped scallions	Cd^2+^	18.6	0.1–3 μM, 5.0–30.0 μM	15 nM	Tap water	Dan et al. (2018)
Melamine, 2,4-difluorobenzoic acid	Cd^2+^	65.5	0–30 μM	0.34 μM	Tap water	Zeng et al. (2022)
Citric acid, o-phosphorylethanolamine	Cd^2+^	8.17	0.5–12.5 μM	0.16 μM	Serum and urine	Lin et al. (2019)
Auricularia auriculawere	Cd^2+^, Hg^2+^	23.57	0–50 μM	101.55 nM, 77.21 nM	Dendrobium	Dai and Peipei Wei. (2021)
L-Cysteine	Hg^2+^	12.6	0.5–20 μM	500 nM	Lake water	Wei et al. (2018)
Sodium citrate, urea	Hg^2+^	67	0.001–5 μM	0.65 μM	Lake water	Ren et al. (2018)
Citric acid, urea, thiourea	Hg^2+^	19.2	0.1–20 μM	62 nM	Tap, river, and mineral water, and canned fish	Tabaraki and Sadeghinejad. (2018)
Citric acid, melamine	Hg^2+^	44	2–14 μM	0.44 μM	Human milk	Pajewska-Szmyt et al. (2020)
L-arginine	Cd^2+^, Hg^2+^	71.6	0–26.8 μM, 0–49.9 μM	0.201 μM, 0.188 μM	Apples and cabbages	This work

The possible application of N-CDs in actual sample detection was evaluated by recovery experiments determined in apples and cabbages. As shown in Table 3, little Cd^2+^ and Hg^2+^ were detected in the digestion solution of apples and cabbages by N-CDs. Then, the standard addition method was performed on the samples spiked with heavy metal ions at different concentrations. Besides, the detected recovery rates for Cd^2+^ and Hg^2+^ were 86.44–109.40% and 86.62–115.32%, respectively, with the RSD below 5%, suggesting the good accuracy and recovery of the method and promised to detect Cd^2+^ and Hg^2+^in real samples.

**TABLE 3:** Analytical results for determination of Cd^2+^ and Hg^2+^ in real samples.

Ions	Sample	Spiked (mg L^−1^)	Found (mg L^−1^)	Recovery (%)	RSD (%)
Cd^2+^	Apple 1	0	—	—	—
	Apple 2	10	9.74	97.40	2.4
	Apple 3	50	48.32	96.64	2.6
	Apple 4	250	241.43	96.57	3.7
	Cabbage 1	0	0	—	—
	Cabbage 2	10	10.94	109.40	3.0
	Cabbage 3	50	43.22	86.44	3.9
	Cabbage 4	250	248.13	99.25	4.5
Hg^2+^	Apple 1	0	—	—	—
	Apple 2	50	49.22	98.44	1.8
	Apple 3	100	115.32	115.32	3.3
	Apple 4	500	433.10	86.62	2.5
	Cabbage 1	0	—	—	—
	Cabbage 2	50	51.14	102.28	3.3
	Cabbage 3	100	112.41	112.41	2.1
	Cabbage 4	500	513.50	102.70	4.8

## Conclusion

In summary, a fast and cheap route to construct N-CDs was obtained using a one-step hydrothermal method with l-arginine as precursors. The prepared N-CDs showed stable, favorable fluorescence properties, and excellent resistance to photobleaching with a high QY of 71.6%. N-CDs exhibited good linear range, sensitivity, tolerance level towards Cd^2+^ and Hg^2+^ ions, and low detection limit. Furthermore, for the detection of heavy metal ions in real food samples, this method has shown high recovery with good reproducibility, which possessed the potential for the rapid and reliable detection of heavy metal ions in agricultural products samples.

## Data Availability

The raw data supporting the conclusions of this article will be made available by the authors, without undue reservation.
