# Supplementary material for: One-step synthesis of highly fluorescent carbon dots as fluorescence sensors for the parallel detection of cadmium and mercury ions
Source: Front Chem. 2022 Sep 30;10:1005231. doi: 10.3389/fchem.2022.1005231 (PMC9563711; doi:10.3389/fchem.2022.1005231)
Supplement: Supplementary file 1 [file DataSheet1.docx]

Supplementary Material


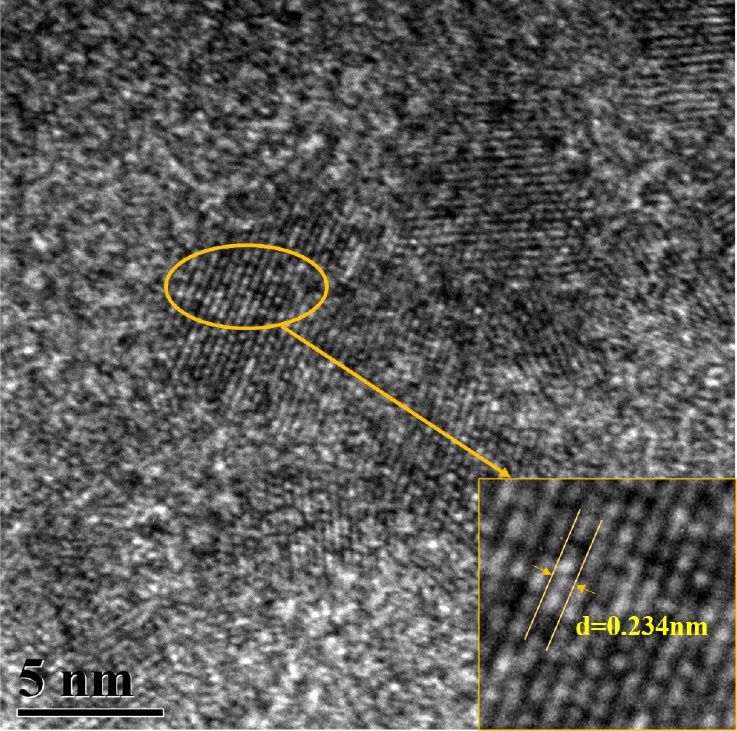


Fig. S1. HRTEM image of N-CDs.


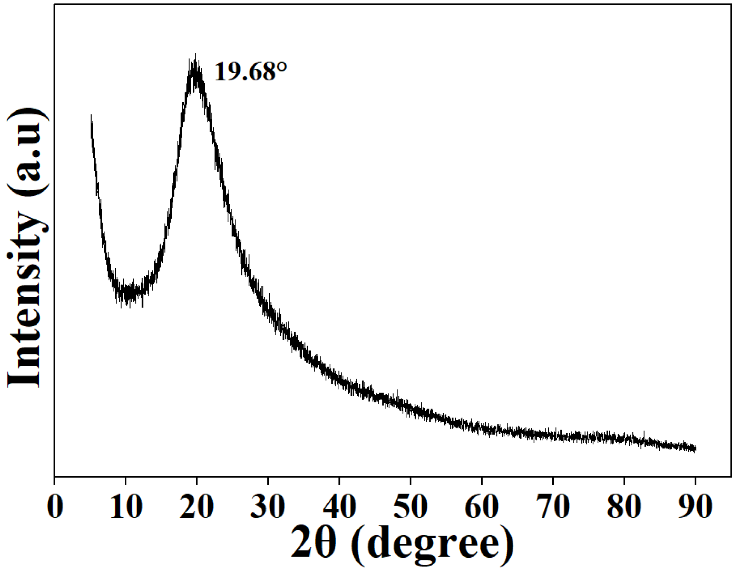


Fig. S2. XRD spectrum of N-CDs.


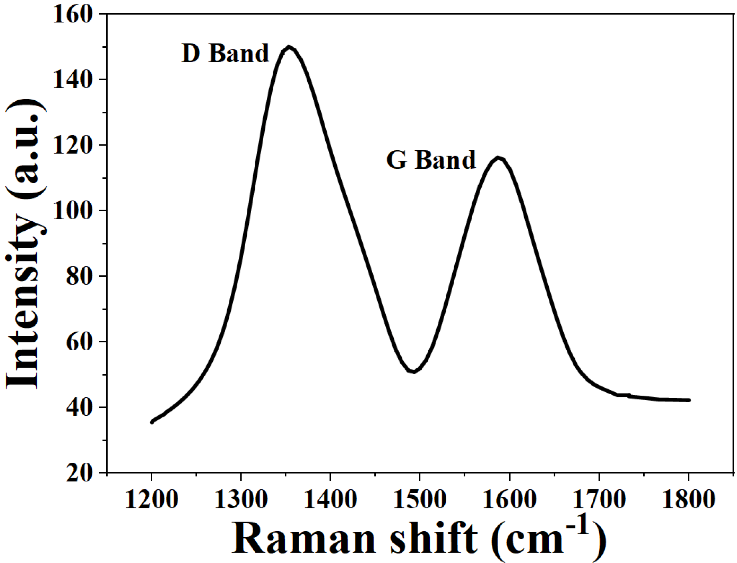


Fig. S3. Raman spectrum of N-CDs.


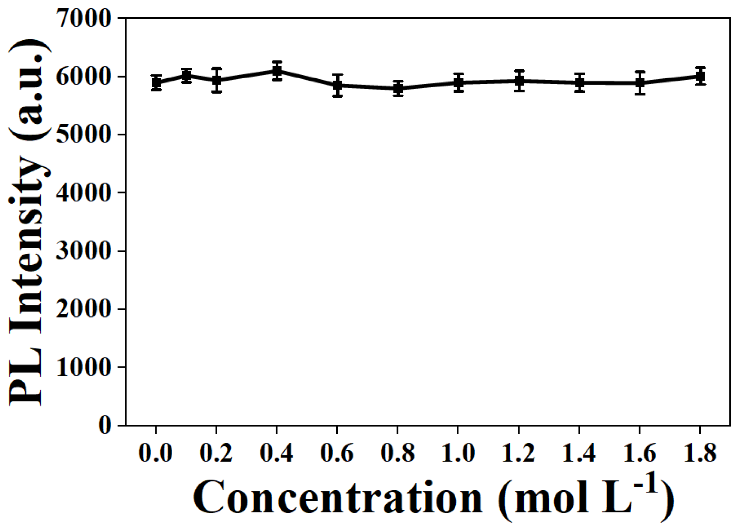


Fig. S4. The effect of salinity on the stability of N-CDs.


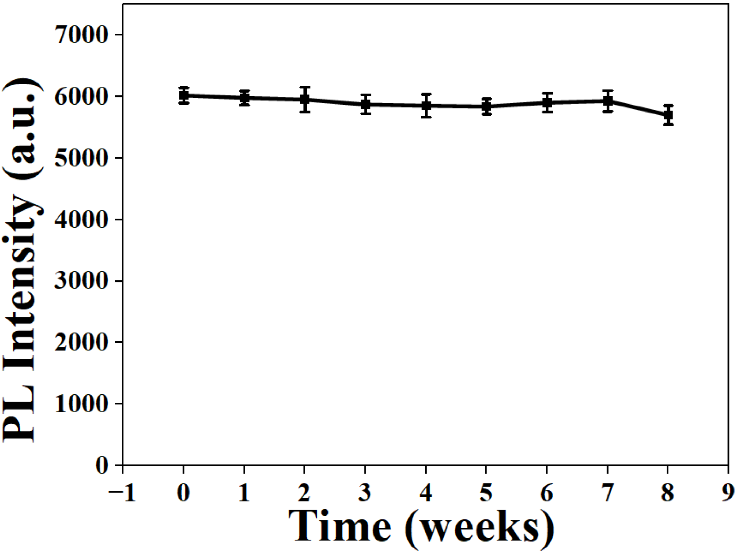


Fig. S5. The effect of storage time on the stability of N-CDs.


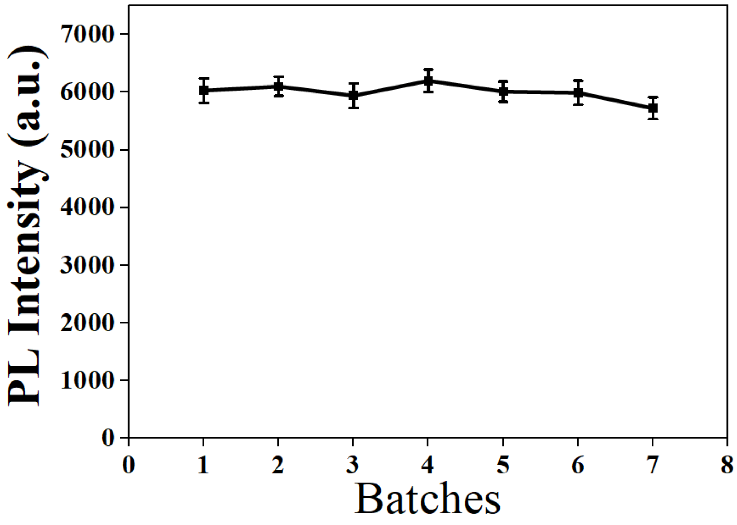


Fig. S6. The effect of different batches on the stability of N-CDs.


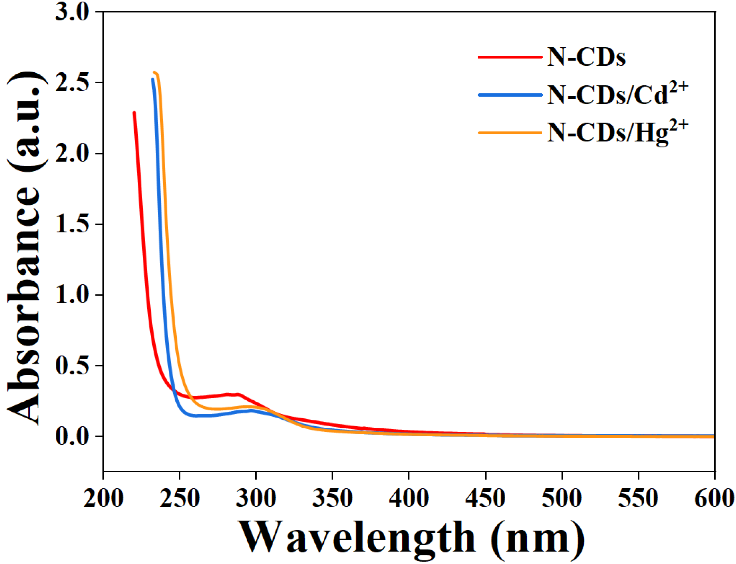


Fig. S7. The UV-vis spectra of N-CDs, N-CDs/Cd^2+^ and N-CDs/Hg^2+^.


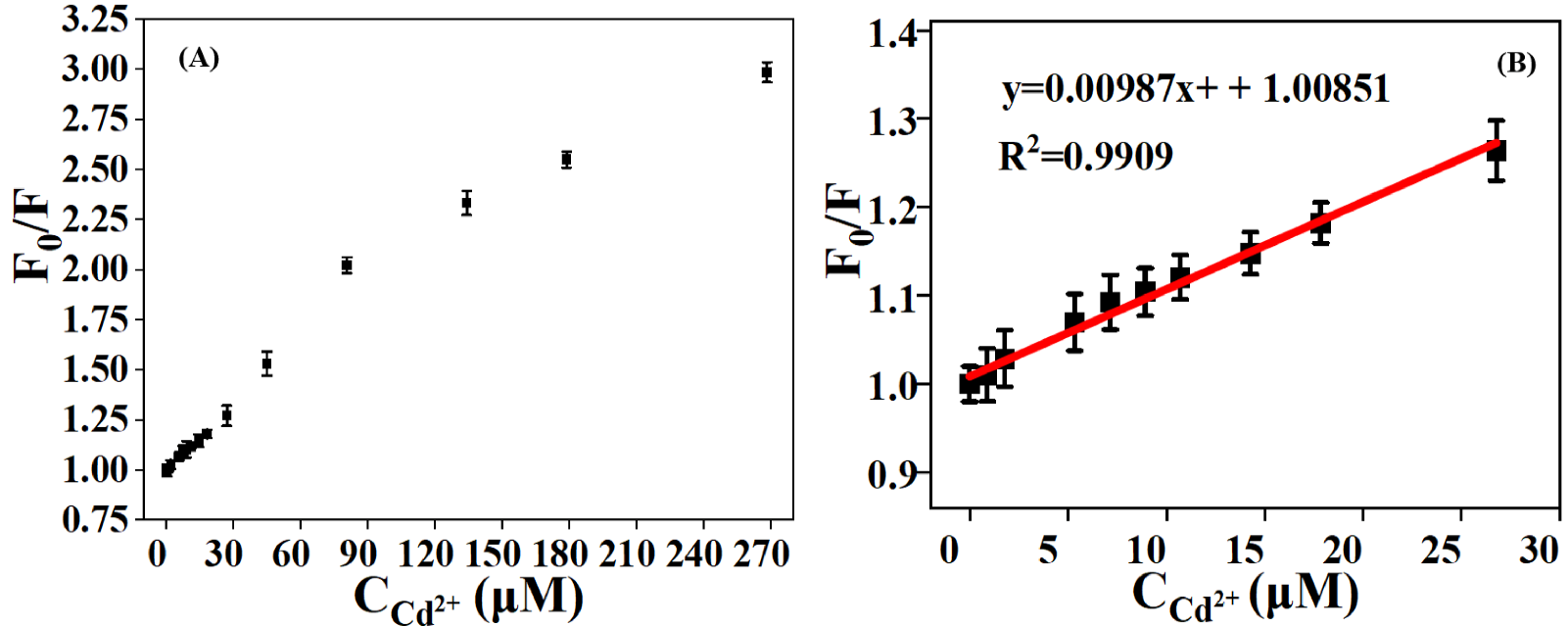


Fig. S8. (A) The Stern-Volmer (SV) plot between F/F_0_ and C_Cd_^2+^; (B) The relationship between F/F_0_ and C_Cd_^2+^, C_Cd_^2+^: 0-26.8 μM.


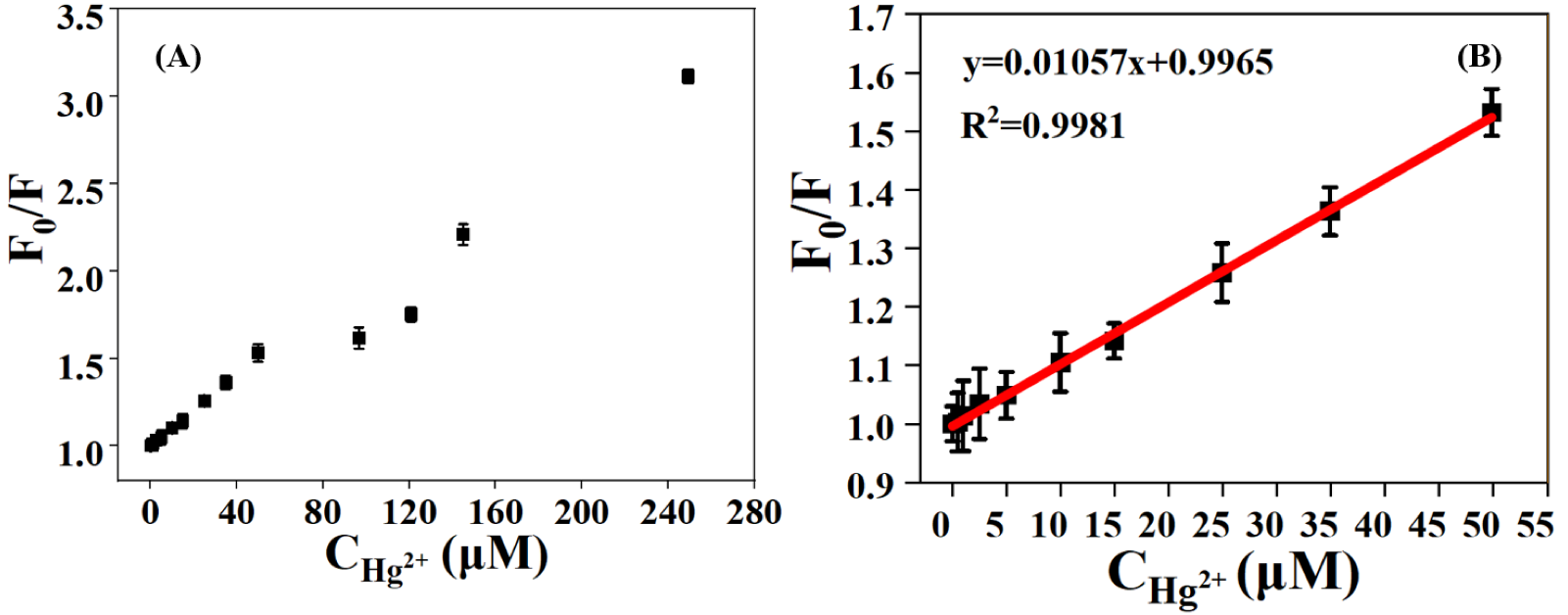


Fig. S9. (A) The Stern-Volmer (SV) plot between F/F_0_ and C_Hg_^2+^; (B) The relationship between F/F_0_ and C_Hg_^2+^, C_Hg_^2+^: 0-49.9 μM.
